# Framework and Schema are False Synonyms: Defining Terms to Improve Learning

**DOI:** 10.5334/pme.947

**Published:** 2023-07-28

**Authors:** Jessica J. Dreicer, Andrew S. Parsons, Tony Joudi, Scott Stern, Andrew P. J. Olson, Joseph J. Rencic

**Affiliations:** 1 Department of Medicine, University of Virginia School of Medicine, Charlottesville, Virginia, US; 2 Fourth-year medical student at the Boston University Chobanian and Avedisian School of Medicine, US; 3 University of Chicago, Chicago, Illinois, US; 4 Departments of Medicine and Pediatrics, University of Minnesota Medical School, Minneapolis, Minnesota, US; 5 Boston University Chobanian and Avedisian School of Medicine, Boston, MA, US

## Abstract

Clinical reasoning is an essential expertise of health care professionals that includes the complex cognitive processes that lead to diagnosis and management decisions. In order to optimally teach, learn, and assess clinical reasoning, it is imperative for teachers and learners to have a shared understanding of the language. Currently, educators use the terms schema and framework interchangeably but they are distinct concepts. In this paper, we offer definitions for schema and framework and use the high-stakes field of aviation to demonstrate the interplay of these concepts. We offer examples of framework and schema in the medical education field and discuss how a clear understanding of these concepts allows for greater intentionality when teaching and assessing clinical reasoning.

## Introduction

In response to calls from national publications [1,2] and the medical literature [3,4] to reduce diagnostic error, medical educators began to teach clinical reasoning earlier and more explicitly in an effort to mitigate cognitive clinical reasoning errors in practice. The majority of these educational initiatives use theory-based clinical reasoning terminology–the *language* of clinical reasoning [5]–in educational venues such as stand-alone courses [6], simulation [7], pre-clerkship curricula [8] and while teaching in the clinical environment [9].

Clinical reasoning curriculum design for undergraduate medical education has benefited from the intentional adaptation of theories and concepts from other disciplines including cognitive psychology [10] and information processing theories (e.g., dual process theory [11], script theory [12], and cognitive load theory [13]). Dual-process theory is foundational to current understanding of clinical reasoning [11]. The dichotomy of pattern recognition versus slow and deliberate thinking serves as a frequent introduction to clinical reasoning. Script theory explains how clinicians organize clinical knowledge into “illness scripts”- clinical findings, risk factors, and pathophysiology- which allow them to match a patient’s presentation to a specific diagnosis [12]. An understanding of cognitive load theory is critical to the way in which we teach clinical reasoning as it deals with the limits of new information that can be integrated into short-term memory and thus provides insights into clinical information processing and diagnostic error [13]. A clear understanding and disambiguation of the underpinning *language* is critical for building undergraduate medical curricula as well as guiding research.

Terms such as dual process theory [14,15], problem representation [14,16], and illness scripts [14,17] are now ubiquitous in clinical reasoning curricula yet even these often lack an agreed upon definition (e.g., are problem representations and summary statements synonyms or distinct entities?) [9,14,18]. Developing commonly understood definitions of this terminology in the medical educational context advances the field by enabling the development, sharing, and evaluation of educational programs for teaching [19], and assessing clinical reasoning [20,21], as well as guiding research.

When teaching clinical reasoning, educators commonly use the terms “schema” and “framework”, though as yet there is no clear consensus definition of these terms in the medical literature and they are often used interchangeably [22,23,24,25,26]. Lack of clarity around this terminology may lead an educator to lack specificity in the design of their teaching session or to fail to consider how their teaching will integrate into what the learner already knows about a topic.

Although they are closely related, we understand schema and framework to be, in fact, distinct entities. In this perspective, we present the original definition of schema from the cognitive psychology literature and offer a definition of framework. Next, we extrapolate lessons about schema and framework from the development of expert performance in the airline industry, a field in which cognitive errors, like in medicine, can cause serious morbidity and mortality. Finally, we discuss schema and framework in the context of clinical reasoning. We posit that clearly defining schema and framework in the context of clinical reasoning will help to advance methods for teaching and learning clinical reasoning and enable future comparative research.

## The Argument for Clearly Defining Schema and Framework

Though the terms scheme, schema, and conceptual framework [22,23,24,25,26] are often used as a synonym for framework in medical education we suggest distinguishing external representations of knowledge organization (i.e., frameworks [27]) from idiosyncratic conscious and unconscious cognitive mental representations of knowledge (i.e., schema). In the most basic sense, human beings use symbols and words to create *frameworks* to share conscious elements of their *schemas* with one another [28].

Without a clear understanding of the distinction between schema and framework, educators may inadvertently teach using frameworks that are not appropriate for the learner’s understanding. For example, trying to teach early undergraduate medical students about the complications of portal hypertension who have no existing schema for the complications of liver disease would be ineffective. Distinguishing schema from framework can assist educators in considering the learner’s existing knowledge organization (schema) and intentionally structure their lesson to convey a specific developmentally-appropriate teaching structure (educator’s framework) that a learner can more readily integrate into their developing schema.

## Defining Schema

The concept of schema likely derives from Immanuel Kant who suggested that the nervous system with its inherent capabilities and limitations shapes human beings’ understanding of the external world/environment through experience [29]. Building on this, subsequent authors provided more practical descriptions, describing schemas as higher order cognitive organizing structures that emerge from interactions between the environment and the central and peripheral nervous systems that mediate human beings’ experiences of the world [30,31]. As such, many schemas are automatically activated (i.e., “instantiated”) by contextual factors with which an individual interacts or has previously interacted. For example, a medical student takes their first history from a patient with abdominal pain but does not have any well-learned approach for asking about abdominal pain so they utilize their existing “history-taking schema” built upon prior conversational experiences but do not ask specifically about onset or radiation of the abdominal pain. Subsequent to this interview, the student attends a lecture on the typical features of different causes of abdominal pain (i.e., illness scripts) as well as a small group session about an approach to history-taking in patients with pain (onset, provoking/palliating factors, quality, radiation, severity, and timing- OPRQRST framework). The next time the learner conducts a patient interview about abdominal pain, they gather a richer history and specifically ask about onset, radiation and severity of their pain as well as typical clinical features associated with different causes.

When one encounters a novel experience for which a related schema exists, the schema affects the interpretation of the novel experience and the novel experience also expands, contracts, or reorganizes the schema [32,33,34,35]. This is demonstrated by the evolution of illness scripts, a particular subtype of schema related to disease knowledge. An early learner starts out with an illness script for a pulmonary embolism learned from a “textbook” that involves a presentation with acute pleuritic chest pain. This illness script evolves as they interact with a patient in real life who presented with pulmonary embolism complaining of exertional dyspnea and subsequently includes pleuritic chest pain and exertional dyspnea. Since illness scripts serve an important function in the “pulmonary embolism diagnosis” schema, the learner will be more likely to consider evaluating for pulmonary embolism in the future with a dyspneic patient.

Schemas are learned through sociocultural experiences and are activated unconsciously in a particular context (e.g., when a learner speaks to patients it is best to avoid medical jargon but when presenting to an attending physician a learner should use medical terminology). Since everyone has a unique collection of prior physical and sociocultural experiences, schemas are idiosyncratic [22]. Parts of schema are unconscious and reflexive; therefore, it is impossible to fully determine which schemas are activated during a series of decisions or behaviors, even by the individual themselves. Additionally, schema activation is influenced by the complexity and familiarity (or lack thereof) of the situation [36].

## Defining Framework

Frameworks are external representations of conscious elements of schema that are developed to clarify and/or simplify relationships between concepts in a specific knowledge domain [27]. Frequently, frameworks are communicated in written or pictorial form. In their optimal form, frameworks are constructed intentionally with a specific goal and audience in mind. For example, an educator might offer a framework for the initial evaluation of an undifferentiated acute kidney injury (Table 1) with the hope that learners will integrate this approach into their schema for evaluating acute kidney injury and thus, have a more thorough, systematic approach when they next encounter this clinical problem.

**Table 1:** Acute Kidney Injury Framework.


PRE-RENAL	INTRARENAL	POST-RENAL

Hypovolemia Systemicvasodilation Renal vasoconstriction	Glomerular insult Tubular insult Interstitial insult Vascular insult	Ureteral obstruction Bladder obstruction Urethral obstruction


**Figure 1:**
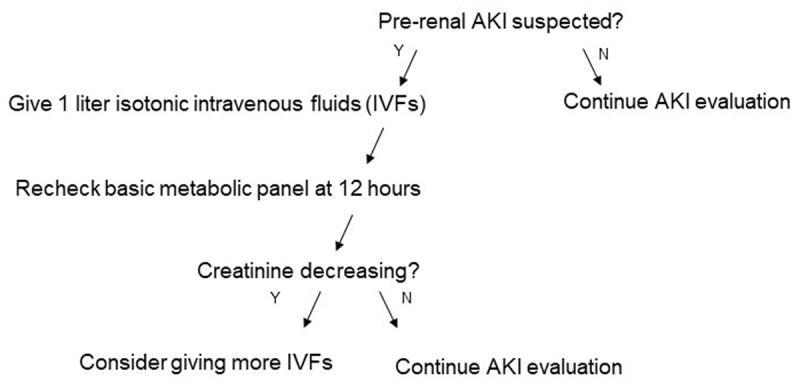
Pre-renal acute kidney injury (AKI) algorithm.

Due to its frequent use, an important subtype of frameworks is the algorithm (Figure 1). An algorithm is a specific type of framework that consists of step-by-step rules or instructions to solve a problem (e.g., if x is present, then y; if x is absent, then z) [37]. Visually, these steps are often represented by branching points and decisions must be made at each branch point as one advances through the algorithm. Algorithms are popular tools in medicine to assist diagnosis and management decisions [38].

## Use of Schema and Framework Outside of Medicine

In aviation, the appropriate use of frameworks and schema are high-stakes because, just as in medicine, human errors can have grave consequences [39]. Pilots are trained through a combination of classroom-based learning and flight simulation [40,41]. Key to this training are frameworks made up of algorithms, which usually exist in the form of checklists [42,43]. Pilots then utilize simulation to deliberately practice these frameworks and develop relevant schema of flight controls, the impact of current flight conditions, and airplane handling characteristics [41,44,45]. In addition to building and refining schema, pilots and their instructors rely on frameworks to enhance performance and use shared mental models [46,47]. These frameworks outline best operating practices and allow for problem-solving in-situ largely derived from structured guidelines designed to ensure uniformity in training and practice. Pilots can then utilize frameworks and schemas when reacting to changes in their environment, especially in situations where schema may not be triggered or may be difficult to access. [48]. During engine failure, a pilot may attempt to restart the engine using a checklist (i.e., framework) while simultaneously determining the most appropriate area for an emergency landing (i.e., utilizing schema) [49]. In this and other flight scenarios, frameworks are utilized to augment schemas that may not be fully internalized or accessible in a given context. However, the importance of individual pilots’ schema must not be under-emphasized. The unconscious activation of schema under pressure is credited with decision making that avoided disasters [41].

## Defining Schema and Framework in Clinical Reasoning

In the context of clinical reasoning, schemas consist of higher order cognitive structures that are triggered or activated in an individual clinician’s mind based on a patient’s presenting problem in a given context [23]. These schemas may include approaches to diagnosing and treating a certain problem [22] and/or information about particular diseases (e.g., illness scripts) as well as how these conditions and their presentations compare and contrast with one another [23]. Schemas are created based on “book” knowledge as well as notable experience with past or recent patient [23]. These schemas are activated because the case at hand shares key features or commonalities with an individual’s knowledge or prior cases that led to their development or refinement [50]. Each clinician’s schema for a given problem in a specific context is unique given their individual clinical and non-clinical experiences. Given the impact of personal experiences on the development of schemas, earlier learners will more often lack schema relevant to the situation at hand. Early learners also have a greater risk of inappropriately developed schema (e.g., extrapolating that ordering a d-dimer is part of the evaluation of all patients with chest pain after seeing a single patient evaluated this way).

**Figure 2:**
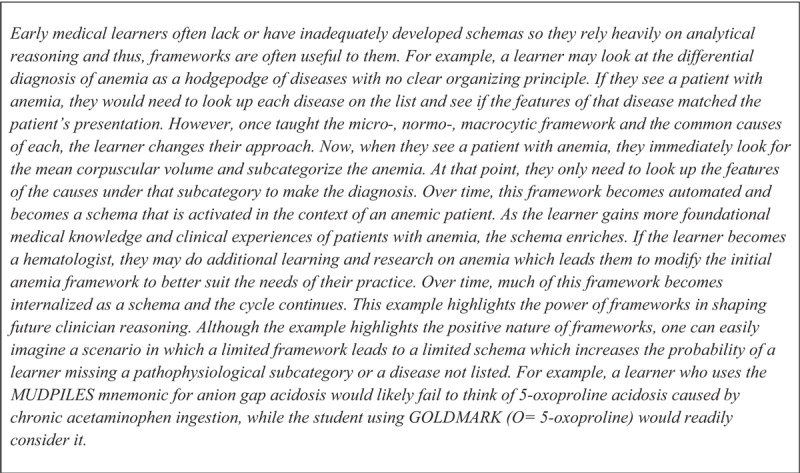
Example of interaction between frameworks and schemas in a medical learner.

In contrast, frameworks provide an organized and simplified structure of complicated, often very detailed, medical knowledge codified outside an individual’s mind. Frameworks that aid in the teaching of diagnostic reasoning (i.e., “diagnostic frameworks”) are often developed through two approaches: 1) using anatomy and pathophysiology to subcategorize a differential diagnosis [51] or 2) focusing on common and deadly (i.e., “can’t miss”) diagnoses [52]. For example, acute kidney injury is often divided into anatomic subcategories (pre-renal, intrarenal, post-renal, see Table 1) to facilitate diagnostic evaluation. Similarly, learners are taught the GOLDMARK (glycolic acids, 5-oxoproline, L-lactic acid, D-lactic acid, methanol, aspirin, renal failure, ketoacidosis) mnemonic for anion gap metabolic acidosis to facilitate recognition of “can’t miss” diagnoses. By organizing a differential diagnosis into subcategories or limiting the number of diseases to be memorized, frameworks can reduce the load on working memory allowing easier recall of the information while also providing a scaffold to organize new knowledge (that will ideally be integrated into the learner’s existing schema, Figure 2). Management frameworks use similar approaches [53,54]. The framework utilized should take into account both the main teaching point (e.g., recognition of life-threatening complications of hyperkalemia and immediate treatment) and the current understanding of the learner (i.e., the learner’s existing schema).

## Use of Schema and Framework in Teaching and Assessing Clinical Reasoning

Educators commonly attempt to organize knowledge into hierarchies and categories (i.e., frameworks) to influence and enhance their learners’ organization and subsequent recall of that information (i.e., schemas) [55,56]. The continued use of frameworks by learners likely leads to changes in neural networks and thus alters an individuals’ schema development. For example, in a subsequent interview about epigastric abdominal pain, our student who has now developed a basic illness script for pancreatitis will inquire about the presence of nausea, vomiting, alcohol use, and specifically asks about radiation of the pain into the back.

**Figure 3:**
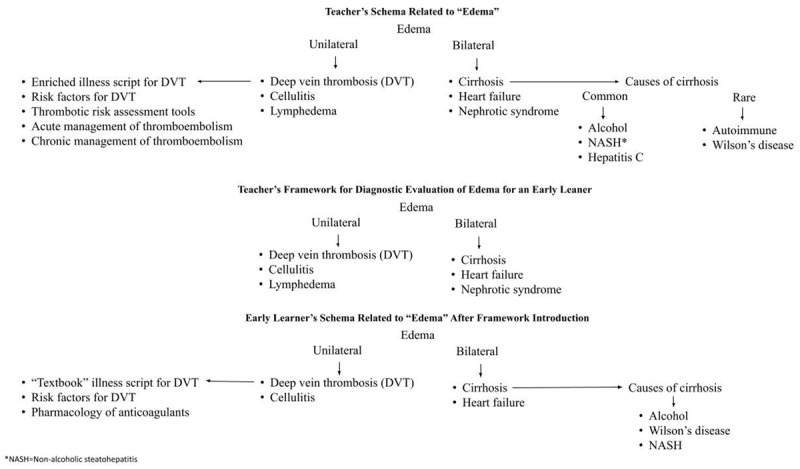
Interaction between learner’s schema and teacher’s framework.

Teachers of clinical reasoning often use frameworks when teaching about a particular topic related to patient care (e.g., “chalk talks”) [26,53,57]. This is, in essence, an effort to shape a learner’s specific schema (e.g., “diagnosis of dyspnea schema,” “management of acute gastrointestinal bleeding schema”). A major goal when teaching clinical reasoning is to enhance the learner’s knowledge (re)organization so they will correctly approach the diagnosis and management of similar patients in the future (Figure 3) [23]. One approach to teach the utilization of frameworks is included in the Supplemental Digital Appendix 1.

As a learner’s clinical reasoning schema become more developed, they will need to consult externalized frameworks less often for common problems. However, in the age of ever-increasing and changing medical knowledge it is imperative to teach learners (through role modeling) to recognize when their schemas are insufficient for making a diagnosis or management decision [58,59]. Situations that should trigger an outside reference include when a diagnosis is not readily apparent [60,61,62] or when the patient does not improve with appropriate treatment [32]. Recognition of the edge of one’s knowledge (i.e., realizing when to seek outside guidance) is crucial to the delivery of excellent clinical care.

Essential questions remain. Does using frameworks to teach clinical reasoning have the desired positive impact on a learner’s schema? More importantly, does teaching this way ultimately improve future patient care? To definitively answer these questions, access to an individuals’ complete schema, including unconscious aspects, would be necessary. For many reasons, complete schema assessment is not possible because of the complexity, idiosyncrasy and the large unconscious component to them. There are, however, ways of starting to explore the answers to these questions by assessing the conscious components of schema. For example, some techniques (e.g., cognitive task analysis) can assist in gaining deeper insights into conscious elements of clinician’s schema and may enhance learners’ performance on procedural based skills [63]. There is evidence that teaching a structured approach to a diagnostic problem improves diagnostic accuracy for early learners [5,64,65], particularly when potentially confounding information is present [66]. Learning via a framework may also lead to enhanced long-term retention of medical information in early learners [67]. The translation of these findings to improving clinical practice is unproven, though given its importance, efforts to study it are worthwhile.

## Future Implications

A shared understanding of the differences between schema and framework will lead to a clearer and deeper understanding of these concepts, and is important for teaching and assessing clinical reasoning in health professions education. In online and social media medical education in particular, schema has become the most common term used for what we define as a framework. Using the terms interchangeably makes it difficult to distinguish between a learner’s schema (i.e., their higher order cognitive structures) and an educator-provided framework (e.g., a structure that divides anemia into micro-, normo-, and macrocytic). Because the cognitive psychology literature clearly defines schema as cognitive structure, we feel strongly that medical educators and researchers should only use the term framework for external knowledge organization and educational aids.

Improved understanding and use of these terms will likely have a direct impact on curricula. We provide an example curriculum using schema and framework in the Supplemental Digital Appendix 1. Clearly, one overarching goal of medical education is to develop a foundation upon which learners can develop rich, effective, and efficient schema for use in clinical practice. Previously described understandings of how expertise develops in clinical reasoning are helpful in creating a logic model upon which to build curricula, starting with frameworks to impart key basic science concepts and then teaching clinical information to help learners build illness scripts and other clinically relevant abstract cognitive structures into their schema [68]. It will also be important to develop approaches and knowledge regarding more and more advanced technologies (such as large language model artificial intelligence programs) that may blur the lines between externalized frameworks and internalized schemas.

Distinguishing schema from framework helps in designing a framework specific to its intended purpose. In addition to helping learners develop their personal, idiosyncratic schema for a specific problem, frameworks can serve as diagnostic checklists or point-of-care resources for learners to perform hypothesis-driven history and physical examination or order laboratory and radiological investigations. This realization should lead educators to carefully define the purpose of any framework that they are developing *a priori*. There is no gold standard framework for a given medical problem and thus, its purpose should determine its structure and content. If the goal is to clarify key subcategories of a complicated problem to help learners begin to develop their schema and keep cognitive load low, then a concise, “big picture” framework might be best. On the other hand, if the purpose of the framework is to provide learners with a point of care resource to aid them in hypothesis-driven data collection, then a more extensive framework may be algorithmic (e.g., “Ask the patient about pleuritic chest pain. If yes, ask the patient about risk factors for developing blood clots.”). Educators should consider explicitly describing the purpose, intended context, and any limitations/caveats of the framework.

One of the most important implications that must be addressed is how the teaching of frameworks informs the development, refinement, and enrichment of schema. We posit in this work that explicitly teaching frameworks (and thus making clear the features that discriminate between anatomical/pathophysiological “buckets” and/or diseases) will aid in the development of better schema in future learning and practice. As cited, there is some evidence to support the relationship between teaching with frameworks and improved diagnostic performance in early learners but this preliminary evidence requires further investigation. We focused our examples on early learners for clarity but postulate that a clear understanding of schema and framework is also relevant to teaching trainees and in continued professional development.

In addition to changing how and what we teach, these ideas have important implications for assessment in medical education. Frameworks, which are common, shared, and concrete means by which to organize clinical knowledge may be assessed in both preclinical and clinical settings through either formal or informal assessment methods, although substantial effort will need to be made to validate and implement such assessment methods such as diagrams that graphically represent relationships between concepts (i.e., concept maps). The assessment of schemas, however, is much less straightforward given their inherent idiosyncrasy. Any effort to assess schemas is incomplete and limited, although not without merit. Like the assessment of illness scripts (as opposed to disease prototype or diagnostic criteria), assessment of schema will require the use of methods that allow learners to express components of their conscious schema and also reveal elements of their unconscious schema. While optimal (or at least workable) methods to do so must still be developed and validated, methods such as concept mapping may prove to be an effective means to gather enough information about learners’ schemas to provide feedback and reorganize knowledge. Clinical teachers may employ methods such as “think aloud” to have learners verbalize conscious elements of their schema for specific problems and then receive feedback on that knowledge organization [69].

The most important and most challenging component we must study is the relationship between teaching (and learning) frameworks and schemas with learner and patient outcomes. It is plausible and logical, but not yet fully known, if teaching frameworks improves schema development for learners. We must evaluate how a learner’s schema and use of frameworks impacts their performance as an independent practicing clinician in terms of diagnostic accuracy, efficiency, and ultimately the quality of care to their patients.

## Additional File

The additional file for this article can be found as follows:

10.5334/pme.947.s1 Supplemental Digital Appendix 1. Example Curricula Using Schema and Frameworks.
