# Supplementary material for: Framework and Schema are False Synonyms: Defining Terms to Improve Learning
Source: Perspect Med Educ. 2023 Jul 28;12(1):294–303. doi: 10.5334/pme.947 (PMC10377745; doi:10.5334/pme.947)
Supplement: Supplemental Digital Appendix 1. — Example Curricula Using Schema and Frameworks. [file pme-12-1-947-s1.pdf]

Supplemental Digital Appendix 1

Example Curricula Using Schema and Frameworks

Traditionally, medical schools' curricula teach students about *diseases* in the preclinical years and often leave diagnostic reasoning to experiential teaching on the clinical wards. This approach is fraught with difficulties. Clinicians are often too busy to spend sufficient time articulating their approaches to students, those approaches may be subconscious and difficult to explain, and of course, not all clinicians are expert diagnosticians. This is further compounded by the randomness of diseases students encounter on their clinical rotations. One needs only look at the frequency of diagnostic error to appreciate that our current approaches are inadequate.

We believe that the first step in the educational process maintains the traditional approach of first teaching about diseases, so that students have a firm understanding of disease pathophysiology, and how that leads to symptoms, signs, and abnormal laboratory and radiological findings. However, we would propose that additional structures are necessary to take students from understanding *diseases* to understanding how to methodically and reliably evaluate *symptoms*. A variety of tools that focus on the approach to symptoms have been employed (including on-line lectures, podcasts, textbooks, interactive on-line cases, pocket reference cards, OSCE examinations and symptom-based courses/curricula.) A few of these are discussed below.

1. A transition course:

Situated between the preclinical and clinical years, a transition course that focuses on teaching approaches to symptoms or commonly encountered lab abnormalities is invaluable. This time period is ideal as students are acutely aware of their need to perform well on the wards and afraid that they are inadequately prepared. Additionally, most students have completed step 1 of the boards and are ready to focus on more relevant clinical matters. A course structure used by one of the authors inserts a two-week all-day curriculum just prior to the clinical rotations. Each half day focuses on one symptom (e.g., chest pain, dyspnea, etc.) or a common lab abnormality (e.g., anemia, acute kidney injury, hyponatremia, etc.). There are also sessions describing ward expectations, teamwork and presenting on the wards. The course outline is shown in figure 1.

Figure 1. Course Outline

Week one

	**Monday**	**Tuesday**	**Wednesday**	**Thursday**	**Friday**
**AM**	Overview to Diagnostic Process	Chest pain	Dyspnea	Syncope	Abdominal pain
**PM**	Headache	Electrocardiogram evaluation	Chest X-rays	Shock	Abnormal Liver Function Tests

Week two

	**Monday**	**Tuesday**	**Wednesday**	**Thursday**	**Friday**
**AM**	Gastrointestinal Bleeding	Acute kidney injury	Headache	Back pain	Presentations
**PM**	Anemia	Hyponatremia	Delirium	Edema	Teamwork

The conceptual framework to teaching these approaches is illustrated below (figure 2). After acquiring some initial data in Phase 1, students and more seasoned clinicians alike will utilize their existing schemas to recognize illness scripts and test those hypotheses. They are reminded to always consider "can't miss" hypotheses. Should this initial phase fail, (as it is more likely to do with less experienced clinicians due to both the vast array of diseases and their multitude of presentations), Phase 2 utilizes well defined and articulated frameworks to teach the students to systematically and intentionally use pivotal points to narrow a broad differential diagnosis into more focused sub-groups. Pivotal points are features of the history, physical exam, or lab tests that *reliably* segregate large differential diagnoses into smaller, more manageable subsets to evaluate. Finally, having narrowed the differential diagnosis into a smaller subset, students are instructed in Phase 3 to focus their data acquisition, to look for risk factors, associated symptoms and signs for each of the *remaining* diseases on their differential in order to rank those hypotheses.

Figure 2

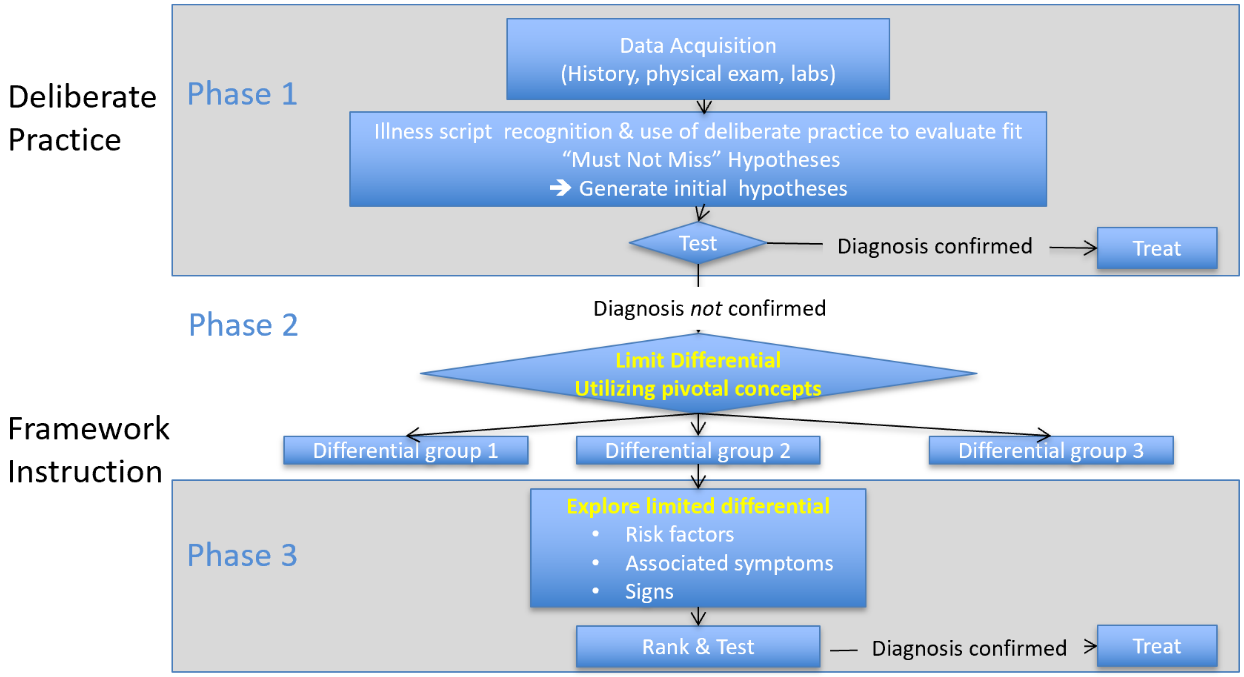

In addition to reinforcing the utility of pivotal points to organize an approach to each day's symptom, the instructors frequently reinforce the concept that many signs and symptoms have limited sensitivity, but may have high specificity. These concepts significantly impact the interpretation of the obtained data.

Each half day is structured as follows (figure 3) and utilizes interactive sessions intended to generate a "need to know".

- Students are given a case with limited information and challenged to submit the additional data they would like to obtain on history, and physical exam. After being given that information they are asked to submit their differential diagnosis and suggested investigations. The case is then left until the end of the session.

- Small student groups then independently work to complete a concept map (framework) for the approach to that symptom. They are provided with a partially completed structured concept map and asked to complete it by providing suggestions for pivotal points (discriminating features) and then place diseases in their appropriate buckets in order to create their own version of symptom specific diagnostic approaches.

- Subsequently, faculty members deliver a symptom-based lecture to describe their evidence based systematic approach (framework) for the symptom in question

- Students then return to their introductory case and algorithms to discuss and answer questions

- Online cases for that particular symptom are then completed by the students facilitating deliberate practice.

- Students can be assessed with multiple choice question tests, OSCE examinations or online simulated cases.

Figure 3

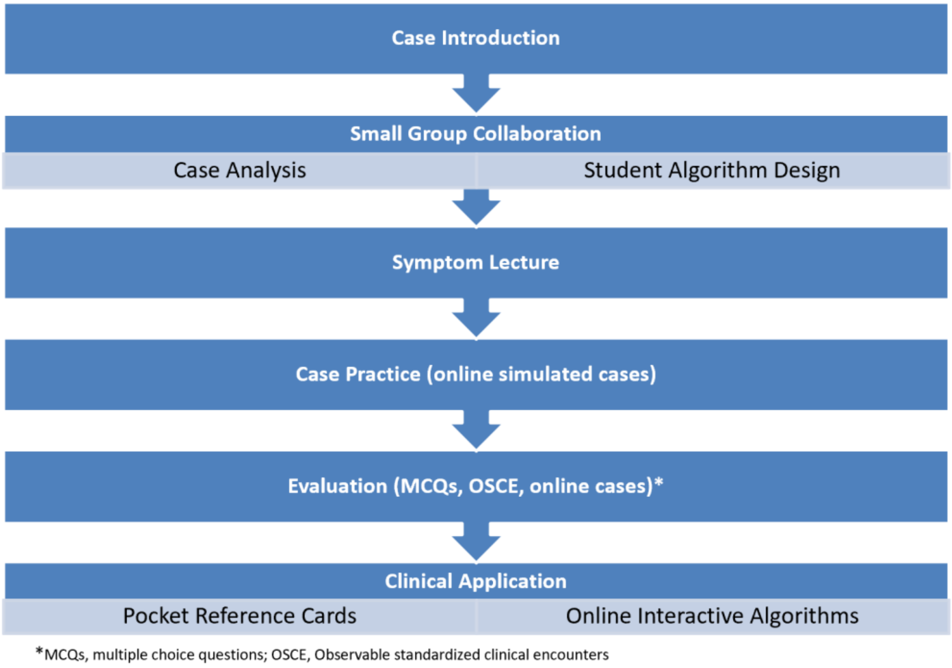

2. Third year interactive symptom case discussions:

During the medicine clerkship, students are instructed to view online symptom-specific lectures prior to participating in interactive faculty facilitated case discussions. These interactive cases help students practice diagnostic approaches presented in the lecture. During the session, the students attempt to diagnose the simulated patient (played by the faculty member.) Students are divided into two teams, to compete against one another as they attempt to make the diagnosis, but avoid unnecessary testing (which is penalized). This creates active engagement (and lively discussions) and allows faculty to immediately provide feedback on both outstanding observations and areas that could be approached more effectively.

3. Urgent Care Senior Elective:

Like the emergency room, urgent care provides an excellent opportunity for students to practice their diagnostic approaches as they evaluate real patients with active problems. One limitation of many such clinical rotations is the busy environment often limits the opportunities for teaching. One of the authors has created an urgent care elective in which *an additional attending* is added to the urgent care clinic solely to work with 2-3 third- or fourth-year students as those students see 2-3 patients each session. The structure is as follows:

- 2-3 weeks in length
- 2-3 students per rotation

Morning Urgent Care Sessions: 6-9 additional patients are scheduled each morning which students see first, and then present to this teaching attending. Since this is an *additional* attending it provides the necessary time to directly supervise the students, evaluate their history, physical exam, differential diagnosis, plan and note (as well as to correct their notes, enter orders etc.)

Afternoon self-directed learning: Each afternoon students and attending all meet again to review their prior and current cases, and determine if a correct or incorrect diagnosis had been made initially, or was still undetermined. Students would be encouraged to research and develop diagnostic approaches to symptoms, study the accuracy of the history, physical exam and diagnostic tests for the diseases under consideration. The afternoon would be utilized to determine what areas should be researched and to review their prior research on the symptom or the diseases in question.

4. Objective Structured Clinical Examinations (OSCEs):

OSCEs have been utilized for many education purposes including evaluating diagnostic reasoning. Students are remotely observed performing a problem focused history and physical exam on a standardized patient. They then meet with the attending to review their findings, differential diagnosis and proposed evaluation. By utilizing very specific criteria, these allow for objective evaluation of students in this arena. In addition, the introduction of this evaluation has helped focus students on studying diagnostic approaches as evidenced by their greater focus on those reading materials. (Unpublished data from Adam Cifu).

5. Simulated online cases:

A variety of simulated online case platforms are now available and being developed. The best of these requires active data acquisition by the students (relevant history and physical exam items), forcing them to create a differential diagnosis and order tests, all of which can be evaluated. Although a good deal of work needs to continue to optimize their performance, they have the promise of 1. Allowing students to utilize deliberate practice in a risk-free environment to hone their diagnostic skills and 2. For the first time, provide the opportunity to ensure that medical students are truly competent in diagnostic approaches. OSCE's, while valuable, are expensive, require a lot of faculty time, and are impossible to scale sufficiently. On-line cases are much easier to scale and could evaluate each student on the evaluation of all of the key symptoms and ensure that they are competent before graduation. This would be the Holy Grail in diagnostic reasoning education.
